# Paclitaxel-coated balloon versus paclitaxel-eluting stent for femoropopliteal arterial disease: A meta-analysis

**DOI:** 10.1097/MD.0000000000041949

**Published:** 2025-03-21

**Authors:** Tingni Tang, Jie Fang, Yongbao Zhang

**Affiliations:** a Becton, Dickinson and Company, Franklin Lakes, NJ; b Department of Aortic and Vascular Surgery Center, State Key Laboratory of Cardiovascular Disease, Fuwai Hospital, National Center for Cardiovascular Diseases, Chinese Academy of Medical Sciences and Peking Union Medical College, Beijing 100037, China.

**Keywords:** femoropopliteal arterial disease, meta-analysis, PCB, peripheral artery disease, PES

## Abstract

**Background:**

Paclitaxel-coated balloon (PCB) and paclitaxel-eluting stent (PES) are widely used in femoropopliteal arterial disease (FPAD), while the comparison of their clinical benefit is inconclusive. This meta-analysis aimed to compare the efficacy between PCB and PES for FPAD.

**Methods:**

Three internet databases were searched for eligible randomized controlled trials (RCTs). Random-effects model was used for pooled clinical outcomes grouped by PCB or PES, following with an indirect comparison. Subgroup analysis was planned according to age, gender, history of smoking, hypertension, and diabetes.

**Results:**

Twenty-five RCTs encompassing 2806 patients were included. There were no significant differences between PCB and PES concerning the incidence of primary patency rate (risk of restenosis [RR]: 0.925; 95% CI: 0.815–1.049; *P* = .222), target lesion revascularization (TLR) (RR: 1.248; 95% CI: 0.798–1.952; *P* = .332), death (RR: 1.130; 95% CI: 0.436–2.930; *P* = .801), restenosis (RR: 1.012; 95% CI: 0.647–1.581; *P* = .959), amputation (RR: 1.000; 95% CI: 0.314–3.181; *P* = 1.000), and thrombosis (RR: 0.240; 95% CI: 0.049–1.180; *P* = .079). Subgroup analysis showed a lower primary patency rate in patients ≥ 70-year-old (RR: 0.703; 95% CI: 0.510–0.968; *P* = .031) and an increased risk of TLR when diabetes proportion was ≥ 40.0% (RR: 1.755; 95% CI: 1.013–3.042; *P* = .045) with PCB. Moreover, PCB might increase mortality in smokers (RR: 1.957; 95% CI: 1.000–3.828; *P* = .050).

**Conclusions:**

Regarding safety, no significant differences was found between PCB and PES. Further large-scale RCTs should be conducted based on the direct comparison results.

## 1. Introduction

Peripheral artery disease (PAD) is 1 of the most common peripheral vessel abnormity that affects more than 200 million people worldwide.^[1]^ It mainly occurs in the lower extremities and is associated with an increased risk of disability, limb loss, and mortality, especially in older adults.^[2,3]^ The most common PAD is femoropopliteal arterial disease (FPAD), which causes intermittent claudication and severe limb ischemia, lowering the quality of life.^[4,5]^ Surgical approaches, conservative treatments, exercise training, and endovascular therapies are widely used for FPAD, but the optimal treatment strategies for FPAD are yet unclear.^[6–9]^

Nowadays, endovascular intervention is widely used in patients with symptomatic FPAD.^[10–12]^ In patients with femoropopliteal stenosis/occlusion lesions < 25 cm, balloon angioplasty can dilate the femoropopliteal lesions without any residues, while suboptimal effects might be observed owing to the lack of scaffolds.^[13]^ Stent implantation has been proven to be a highly successful procedure, but in FPAD patients, the risks of stent fracture, thrombosis, and restenosis are high.^[14–16]^ During the past decade, compared with uncoated balloons or bare-metal stents, a drug-device combination approach using paclitaxel has been developed to reduce the risk of restenosis (RR).^[17]^ A meta-analysis by Feng et al including 17 randomized controlled trials (RCTs) revealed that compared to uncoated balloon angioplasty for FPAD, paclitaxel-coated balloon (PCB) exerted better effects on minimal luminal diameter, late lumen loss, primary patency, restenosis, target lesion revascularization (TLR), and major adverse events.^[18]^ Hajibandeh et al performed a meta-analysis consisting of 2 RCTs and 4 retrospective cohort studies and found that paclitaxel-eluting stent (PES) was associated with an increased patency rate and a lower risk of reintervention compared with bare-metal stent.^[19]^ Still, the curative effects of PCB and PES on FPAD patients remain controversial. Therefore, we performed an indirect comparison meta-analysis to assess the effects of PCB vs PES for treating patients with FPAD.

## 2. Methods

### 2.1. Data sources, search strategy, and selection criteria

This study was performed in accordance with the preferred reporting items for systematic reviews and meta-analysis (PRISMA) statement guidelines. Ethical approval and informed consent were not required because this study is a meta-analysis. The protocol of this meta-analysis has not been registered in PROSPERO. The publication language and status were not restricted, but the studies designed as RCT and investigated the treatment effects of PCB or PES on FPAD patients were eligible for this meta-analysis. PubMed, Embase, and the Cochrane Library were systematically searched for eligible trials from inception until January 2025, using the following search terms: “drug-eluting balloon,” “drug-eluting stents,” “femoral,” “femoral artery,” “femoropopliteal,” “infrainguinal,” and “RCTs.” We further consulted ClinicalTrials.gov (US NIH) to retrieve studies that had already been completed but not yet published. The reference lists of the relevant studies were also checked manually to identify any new eligible studies.

The literature database search and study selection processes were performed independently by 2 reviewers according to a standardized approach, and inconsistencies between them were settled by mutual discussions. A study was included if the following inclusion criteria were fulfilled: patients: FPAD; Intervention: PCB or PES; outcome: at least 1 of the outcomes including primary patency rate, TLR, death, restenosis, amputation, and thrombosis was reported; study design: RCT.

### 2.2. Data collection and quality assessment

Two reviewers independently abstracted the following items: first authors’ name, publication year, country, center, sample size, mean age, male (%), hypertension (%), diabetes mellitus (%), post-procedural antiplatelet therapy, intervention, control, follow-up duration, and reported outcomes. Furthermore, the quality of individual trials was assessed using the Jadad scale, which was based on randomization, blinding, allocation concealment, withdrawals, dropouts, and the use of intention-to-treat analysis.^[20]^ The Jadad scale was 0 to 5, and a study with a 5-score was considered optimal quality.^[20]^ Any disagreement between reviewers regarding data collection and quality assessment was settled by an additional reviewer by referring to the original article.

### 2.3. Statistical analysis

The pooled incidence of primary patency rate, TLR, death, restenosis, amputation, or thrombosis in patients treated with PCB or PES was computed using the metaprop command in STATA software (version 10.0; Stata Co., College Station). The pooled results were evaluated by a random-effects model that considered the underlying variations among the included trials. The heterogeneity across included trials was assessed using *I*^2^ and *Q* statistics, and significant heterogeneity was defined as *I*^2^ > 50.0% or *P* < .10. An indirect comparison between PCB and PES was estimated based on the pooled incidences of investigated outcomes, and relative risks with 95% confidence intervals were calculated. Subgroup analyses for reported outcomes were performed concerning age, male proportion, smoking status, hypertension, and diabetes. Both qualitative and quantitative approaches were used to assess publication bias, including funnel plots and Egger and Begg tests.^[21]^ The inspection level for pooled conclusions was 2-sided, and *P* < .05 was regarded as statistically significant.

## 3. Results

### 3.1. Literature search

**Figure 1.**
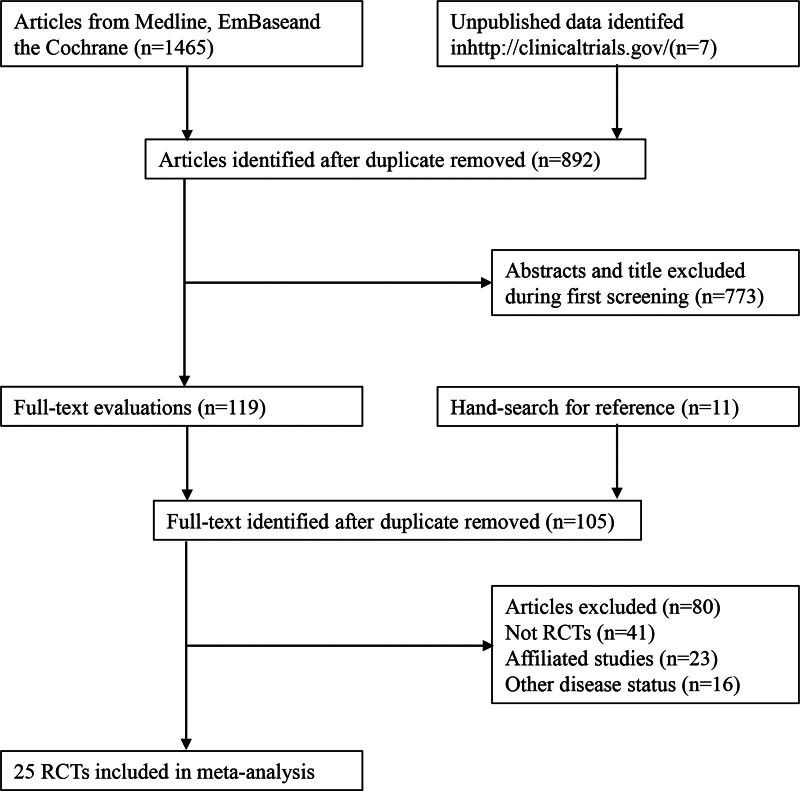
Preferred reporting items for systematic reviews and meta-analysis flowchart for the literature search and study selection process.

The electronic search of the databases retrieved 1472 potential studies, of which 892 articles were retained after duplicates were removed. Subsequently, 773 studies were excluded because these articles reported irrelevant topics. The remaining 119 studies were subjected to full-text evaluations, following which 80 studies were removed because they were not RCTs (n = 41), were affiliated studies (n = 23), or reported other diseases (n = 16). Reviewing the unpublished titles from ClinicalTrials.gov and the reference lists did not yield new studies that fulfilled the inclusion criteria. Finally, the remaining 25 RCTs were selected for this meta-analysis (Fig. 1).^[22–46]^

### 3.2. Study characteristics

The 25 included trials encompassed 2806 patients with FPAD. The baseline characteristics of studies and patients are shown in Table 1. Interestingly, 22/25 studies were multicenter trials, and the remaining 3 studies were single-center trials. Twenty-one studies reported the treatment effects of PCB, and 6 studies reported the effects of PES on patients with FPAD. Moreover, 17 RCTs were of high quality, and the Jadad scores for these studies were 4 or 5. The remaining 8 studies had a low to moderate quality, and the Jadad score was 3.

**Table 1:** Characteristics of included studies.

Study	Country	Center	Intervention	Control	Sample size		Age		Male (%)		Smoking status (%)	Hypertension (%)		DM (%)		Post-procedure antiplatelet therapy	Follow-up (mo)	Outcomes	Quality
					*I*	*C*	*I*	*C*	*I*	*C*		*I*	*C*	*I*	*C*				
FemPac 2008	Germany	Multicenter	PCB	Uncoated balloon group	45	42	67.3	70.2	60	60	47	78	81	40	55	Aspirin (100 mg/d), Clopidogrel (75 mg/d)	24	TLR, death, restenosis, amputation	4
Thunder 2008	Germany	Multicenter	PCB	Standard uncoated balloons and standard nonionic contrast medium	48	54	69	68	65	63	23	79	83	50	22	Aspirin (100 mg/d), Clopidogrel (75 mg/d)	24	TLR, death, restenosis, amputation	5
Pacifier 2012	Germany	Multicenter	PCB	Uncoated balloon	44	47	71	71	59	64	48.8	65.9	66	43.2	27.7	NA	12	TLR, death, restenosis, amputation	4
Debellum 2014	Italy	Single-center	PCB	Conventional angioplasty balloon	25	25	66.5	66.8	76	72	68	76	60	52	36	Aspirin (100 mg/d), Clopidogrel (75 mg/d)	12	TLR, death, amputation, thrombosis	4
LEVANT I 2014	Germany	Multicenter	PCB	Uncoated balloon	49	52	67	70	69	58	31	96	87	45	50	Clopidogrel (75 mg/d)	24	TLR, death, amputation, thrombosis	4
Debate-ISR 2014	Italy	Single-center	PCB	Balloon angioplasty	44	42	74	76	72.7	55	31.8	88.6	91	100	100	Aspirin (100 mg/d), Clopidogrel (75 mg/d)	12	TLR, death, restenosis, amputation	3
Biolux P-I 2015	Germany	Multicenter	PCB	Balloon angioplasty	30	30	70.1	71	56.7	57	63.3	76.7	70	36.7	30	NA	12	TLR, death, restenosis, amputation, thrombosis	5
In. pact SFA 2015	USA	Multicenter	PCB	Percutaneous transluminal angioplasty	220	111	67.5	68	65	68	38.6	91.4	88	40.5	49	Aspirin (81–325 mg/d), Clopidogrel (75 mg/d)	12	TLR, death, thrombosis	5
Levant 2 2015	USA	Multicenter	PCB	Standard Angioplasty Balloon	316	160	67.8	69	61.1	67	35.1	89.2	88	43.4	42	Aspirin (75–100 mg/d), Clopidogrel (75 mg/d)	12	TLR, death, restenosis, amputation, thrombosis	5
Biolux P-II 2015	Germany	Multicenter	PCB	Percutaneous transluminal angioplasty.	36	36	72.9	70	75	83	55.6	86.1	86	61.1	72	Aspirin (100–325 mg/d), Clopidogrel (75 mg/d)	12	TLR, death, amputation, thrombosis	5
Pacuba 2016	Austria	Multicenter	PCB	Percutaneous transluminal angioplasty.	35	39	68.1	68	57	59	52	79	79	52	38	Aspirin (100 mg/d), Clopidogrel (75 mg/d)	12	TLR,	4
Illumenate 2017	Germany and Austria	Multicenter	PCB	Percutaneous transluminal angioplasty.	222	72	67	69	72	68	89	78	83	37	36	Aspirin, Clopidogrel	12.0 and 24.0	TLR, death, amputation	4
Ranger SFA 2017	Europe	Multicenter	PCB	Any nondrug-coated balloon available in an appropriate size.	71	34	68	67	75	68	41	82	76	39	35	Aspirin (75 mg/d), Clopidogrel (75 mg/d)	12	TLR,	3
Consequent 2017	Germany	Multicenter	PCB	Uncoated balloon	78	75	68.2	68	60.3	76	46.2	76.9	80	34.6	39	Aspirin (100 mg/d), Clopidogrel (75 mg/d)	24	TLR, death, restenosis	4
Isar-Pebis 2017	Germany	Multicenter	PCB	Balloon angioplasty	36	34	70	68	67	70	58	92	88	33	35	Aspirin (100 mg/d), Clopidogrel (75 mg/d)	24	TLR, death, amputation, thrombosis	3
Biopac 2018	Poland	Multicenter	PCB	Uncoated balloon	33	33	65.2	66	90.9	70	NA	NA	NA	33.3	33	NA	12	TLR, death, amputation	3
AcoArt I 2018	China	Multicenter	PCB	Uncoated balloon percutaneous transluminal angioplasty	100	100	65.9	66	73	74	29	62	72	54	57	Aspirin, Clopidogrel	12.0 and 24.0	TLR, death, amputation	4
EffPac 2020	Germany	Multicenter	PCB	Conventional balloon angioplasty	85	86	68	68	60	60	40	87	73	36	35	Aspirin, Clopidogrel	24	TLR, restenosis	3
Copa cabana 2020	Germany and Switzerland	Multicenter	PCB	Uncoated balloon	47	41	68.3	68	55	63	30	81	73	43	46	Aspirin (100 mg/d), Clopidogrel (75 mg/d)	24	TLR, death, restenosis, amputation	4
Drastico 2019	Italy	Single-center	PCB	PES	96	96	74.7	74.2	51	76	50	76	80	70	60	NA	12	TLR, death, restenosis, amputation	4
Real PTX 2019	Germany and Belgium	Multicenter	PCB	PES	75	75	68.2	69.5	60	76	42.7	78.7	81.3	34.7	30.7	Aspirin, Clopidogrel	12	TLR, death, amputation	3
Zilver PTX 2011	Europe, USA, and Canada	Multicenter	PES	Percutaneous transluminal angioplasty	236	238	67.9	68	65.7	64	86.4	89	89	49.2	49	Clopidogrel	12.0 and 24.0	TLR, death, amputation	3
Imperial 2018	Europe, USA, Japan and Canada	Multicenter	PES	PTX	309	156	68.5	67.8	66	67	35	82	85	42	44	Aspirin (100 mg/d), Clopidogrel (75 mg/d)	12	TLR, death, amputation, thrombosis	5
Battle 2020	France	Multicenter	PES	Misago	86	85	71	68	72	73	23	69	61	48	26	Clopidogrel (75 mg/d)	12.0 and 24.0	TLR, death, restenosis, amputation, thrombosis	4
Zilverpass 2020	Europe	Multicenter	PES	Bypass	113	107	69.6	68	69	76	69	65.5	81	27.4	32	Aspirin, Clopidogrel	12	TLR, death, amputation, thrombosis	3

C = control group, I = intervention group, NA = not applicable, PCB = paclitaxel-coated balloon, PES = polymer-coated, paclitaxel-eluting stent, PTX = polymer-free, paclitaxel-coated PTX stent, TLR = target lesion revascularization.

### 3.3. Primary patency rate

The effect of PCB and PES on primary patency rate in FPAD patients was reported in 12 and 6 trials, respectively. The pooled primary patency rate data for patients treated with PCB and PES were 74.7% (95% CI: 66.1%–83.3%; *P* < .001) and 80.8% (95% CI: 76.7%–84.8%, *P* < .001), respectively (Fig. S1, Supplemental Digital Content, http://links.lww.com/MD/O594). Moreover, significant heterogeneity was detected in the primary patency rate after using PCB (*I*^2^ = 92.7%; *P* < .001) or PES (*I*^2^ = 57.6%; *P* = .038). Furthermore, there was no significant difference in this treatment outcome when comparing PCB vs PES (RR: 0.925; 95% CI: 0.815–1.049; *P* = .222). Subgroup analysis demonstrated that PCB was associated with a lower primary patency rate compared to PES when the mean age of patients was ≥70 years (Table S1, Supplemental Digital Content, http://links.lww.com/MD/O595). A significant publication bias was detected to estimate the primary patency rate (*P*-value for Egger: .007; *P*-value for Begg: .015; Fig. S2, Supplemental Digital Content, http://links.lww.com/MD/O594).

### 3.4. TLR

**Figure 2.**
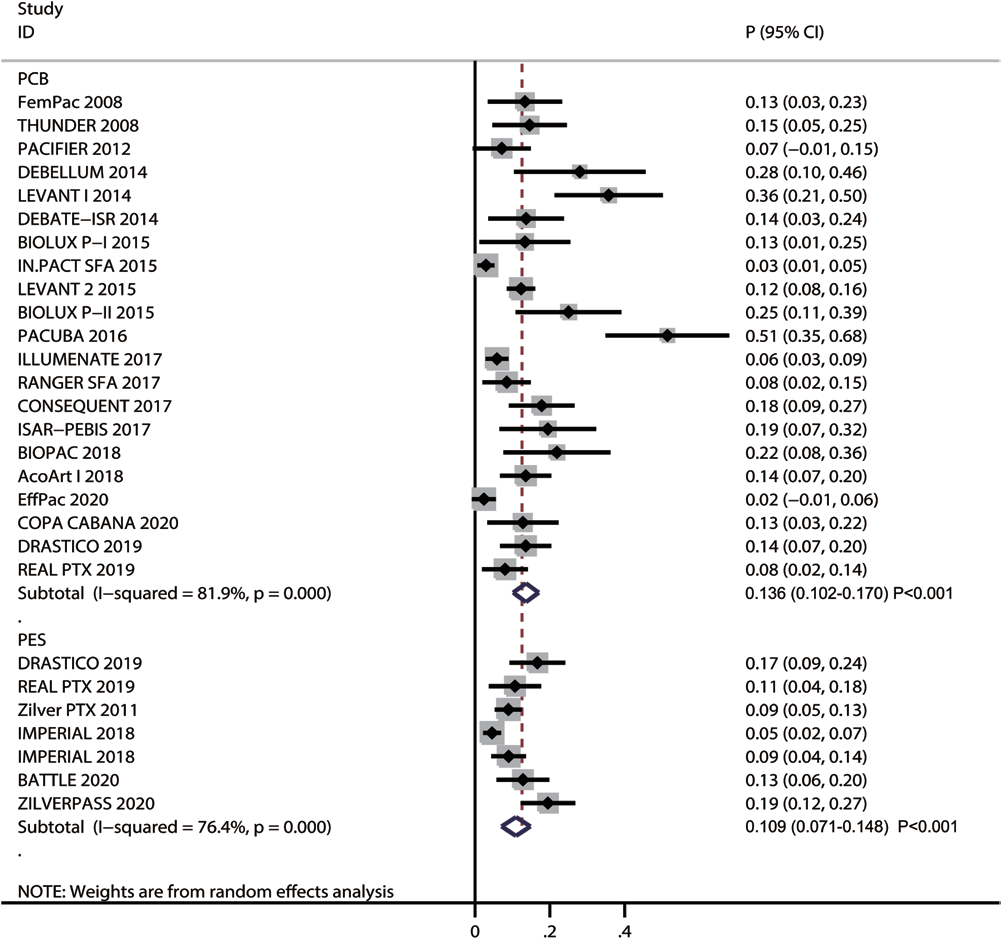
Forest plot with pooled risk for TLR after-treatment categorized by PCB and PES. PCB = paclitaxel-coated balloon, PES = polymer-coated, paclitaxel-eluting stent, TLR = target lesion revascularization.

All included trials reported the treatment effects of PCB and PES on the risk of TLR in patients with FPAD. The pooled risk for TLR after PCB and PES was 13.6% (95% CI: 10.2%–17.0%; *P* < .001) and 10.9% (95% CI: 7.1%–14.8%; *P* < .001), respectively (Fig. 2). Significant heterogeneity was noted among included studies on TLR after PCB (*I*^2^ = 81.9%; *P* < .001) or PES (*I*^2^ = 76.4%; *P* < .001), while no significant difference in TLR risk between PCB and PES was observed (RR: 1.248; 95% CI: 0.798–1.952; *P* = .332). Subgroup analysis found that PCB was associated with an increased risk of TLR compared with PES when diabetes proportion was ≥ 40.0% (Table S2, Supplemental Digital Content, http://links.lww.com/MD/O595). The publication bias for TLR was significant when combining the 2 groups (*P*-value for Egger: <.001; *P*-value for Begg: <.001; Fig. S3, Supplemental Digital Content, http://links.lww.com/MD/O594).

### 3.5. Death

**Figure 3.**
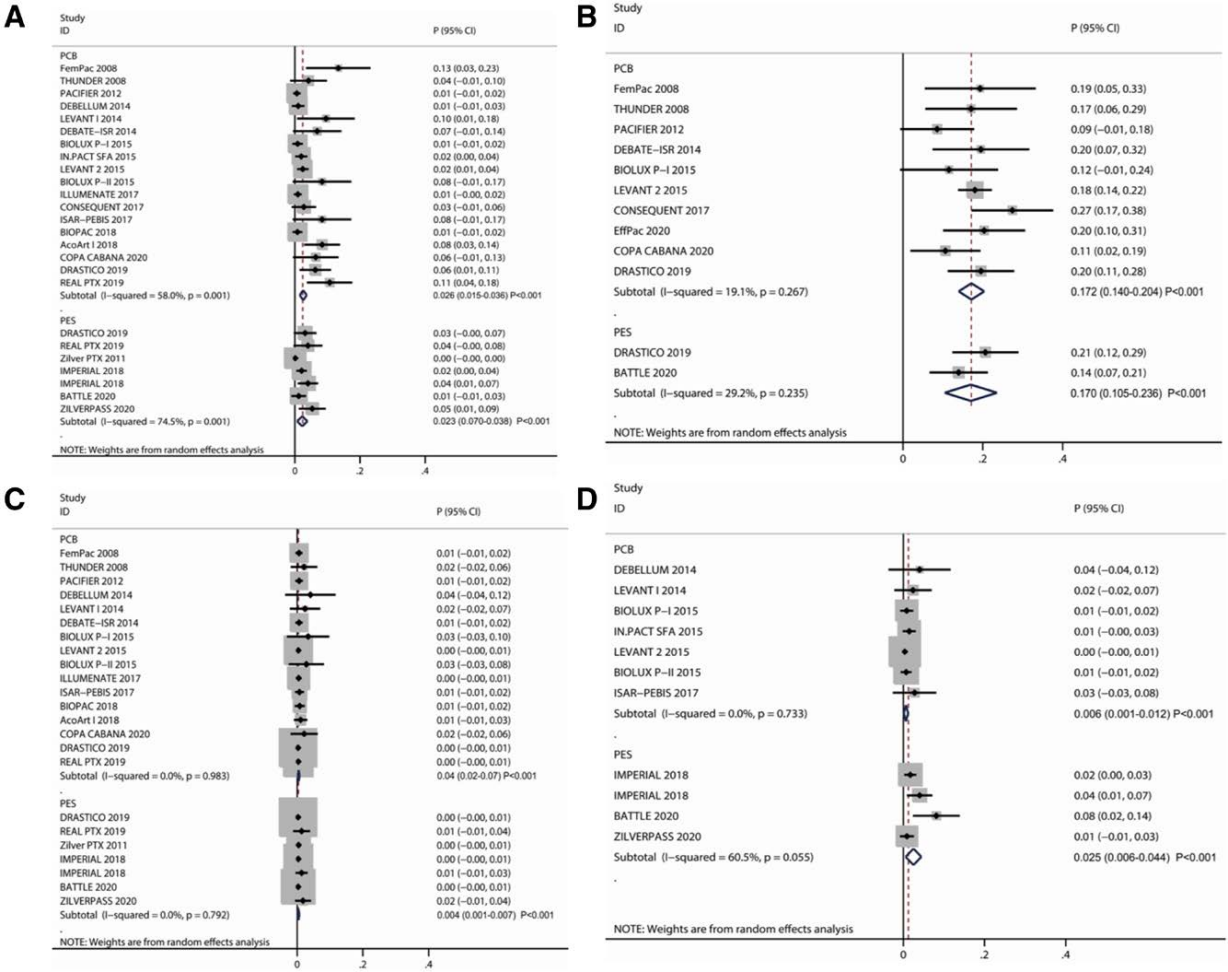
Forest plot with pooled after-treatment risk by PCB and PES for (A) death; (B) restenosis; (C) amputation; and (D) thrombosis. PCB = paclitaxel-coated balloon, PES = polymer-coated, paclitaxel-eluting stent.

The number of trials that reported the effects of PCB and PES on death were 18 and 6, respectively. The pooled death rate after PCB and PES was 2.6% (95% CI: 1.5%–3.6%; *P* < .001) and 2.3% (95% CI: 0.7%–3.8%; *P* = .005), respectively (Fig. 3A). A significant heterogeneity was observed across the studies on PCB (*I*^2^ = 58.0%; *P* = .001) and PES (*I*^2^ = 74.5%; *P* = .001). PCB was not associated with higher death than PES (RR: 1.130; 95% CI: 0.436–2.930; *P* = .801). Subgroup analysis indicated that PCB was associated with increased mortality compared to PES when current smoking was < 60.0% (Table S3, Supplemental Digital Content, http://links.lww.com/MD/O595). A significant publication bias was recorded for death (*P*-value for Egger: <.001; *P*-value for Begg: .001; Fig. S4, Supplemental Digital Content, http://links.lww.com/MD/O594).

### 3.6. Restenosis

The correlations of restenosis risk with PCB and PES were reported in 10 and 2 studies, respectively. The pooled risk for restenosis after PCB and PES was 17.2% (95% CI: 14.0%–20.4%; *P* < .001) and 17.0% (95% CI: 10.5%–23.6%; *P* < .001), respectively (Fig. 3B). No significant heterogeneity was detected among studies on PCB (*I*^2^ = 19.1%; *P* = .267) and PES (*I*^2^ = 29.2%; *P* = .235), and no significant difference was observed between PCB and PES for the RR (RR: 1.012; 95% CI: 0.647–1.581; *P* = .959). Also, subgroup analysis did not find any difference between PCB and PES for the RR (Table S4, Supplemental Digital Content, http://links.lww.com/MD/O595), and no evidence of publication bias was noted (*P*-value for Egger: .938; *P*-value for Begg: .631; Fig. S5, Supplemental Digital Content, http://links.lww.com/MD/O594).

### 3.7. Amputation

The effects of PCB or PES on the risk of amputation were examined in 16 and 6 trials, respectively. The pooled risk for amputation after PCB and PES was 0.4% (95% CI: 0.2%–0.7%; *P* = .001) and 0.4% (95% CI: 0.1%–0.7%; *P* = .015), respectively (Fig. 3C). No significant cross-study heterogeneity was observed after PCB (*I*^2^ = 0.0%; *P* = .983) and PES (*I*^2^ = 0.0%; *P* = .792). No significant difference was detected between PCB and PES for the risk of amputation (RR: 1.000; 95% CI: 0.314–3.181; *P* = 1.000) in all subgroups (Table S5, Supplemental Digital Content, http://links.lww.com/MD/O595). However, a potentially significant publication bias was detected for amputation (*P*-value for Egger: <.001; *P*-value for Begg: <.001; Fig. S6, Supplemental Digital Content, http://links.lww.com/MD/O594).

### 3.8. Thrombosis

The effects of PCB or PES on the risk of thrombosis were discussed in 7 and 3 trials, respectively. The pooled risk for thrombosis after PCB and PES was 0.6% (95% CI: 0.1%–1.2%; *P* < .001) and 2.5% (95% CI: 0.6%–4.4%; *P* < .001), respectively (Fig. 3D). We also noted potentially significant heterogeneity among studies of thrombosis after PES (*I*^2^ = 60.5%; *P* = .055), while no evidence of heterogeneity for thrombosis was detected after PCB (*I*^2^ = 0.0%; *P* = .733). There was no significant difference between PCB and PES for the risk of thrombosis (RR: 0.240; 95% CI: 0.049–1.180; *P* = .079) in all subgroups (Table S6, Supplemental Digital Content, http://links.lww.com/MD/O595). However, we noted significant publication bias for thrombosis (*P*-value for Egger: .001; *P*-value for Begg: .029; Fig. S7, Supplemental Digital Content, http://links.lww.com/MD/O594).

## 4. Discussion

The drug-device combination approach has been widely used for FPAD, while no meta-analysis was performed to compare the treatment effects between PCB and PES for patients with FPAD. This large-scale, quantitative meta-analysis included 2806 patients with FPAD from 25 RCTs, encompassing a wide range of patients’ characteristics, and the pooled conclusions were robust. This study did not detect any significant differences between PCB and PES concerning the incidence of primary patency rate, TLR, death, restenosis, amputation, and thrombosis. Subgroup analyses found that compared to PES, PCB was associated with a low primary patency rate in the subgroup of patients aged ≥ 70 years. Moreover, PCB was associated with an increased risk of TLR when diabetes proportion was ≥ 40.0%. Furthermore, a higher death rate was observed after PCB for patients with current smoking < 60.0% compared to those after PES.

Wang et al performed a meta-analysis based on 2 RCT and 5 cohort studies comparing clinical outcomes (12-month all-cause mortality, 12-month primary patency, 12-month freedom from TLR, and 12-month amputation-free survival) of DES and DCB in patients with lower extremity PADs were eligible for our study.^[47]^ Results suggested that no significant difference in 12-month all-cause mortality, primary patency and freedom from TLR between DES and DCB. However, an indirect comparison approach was used in this study. This approach was based on the pooled incidences of investigated outcomes after using PCB or PES, and hence, the heterogeneity was substantial across the included trials. This phenomenon could be ascribed to the patients’ characteristics, intervention, background therapies, follow-up duration, and imbalance in the study quality between PCB and PES. Therefore, the conclusions of this study should be recommended cautiously in clinical practice, and further high-level evidence needs to be gathered to compare the treatment effects between PCB and PES for patients with FPAD.

The present study did not find any significant difference between PCB and PES in the incidence of primary patency rate, TLR, death, restenosis, amputation, and thrombosis. Two included RCTs reported the comparative treatment effects between PCB and PES for patients with FPAD.^[31,32]^ The DRASTICO study suggested that PES was associated with a larger post-procedural minimal luminal diameter and lower incidence of residual dissection than PCB, while no significant difference was observed between groups for the RR and TLR.^[31]^ Similarly, the REAL PTX study did not detect any differences between PCB and PES concerning the incidences of primary patency rate, TLR, and death in patients with FPAD.^[32]^ However, the treatment effects of PCB and PES were restricted to long and totally occluded lesions, which in turn was associated with an increased risk of patency failures. The femoropopliteal arterial lesion length and total occlusion are independent predictors of restenosis. The background use of devices for vessel preparation also played a significant role in the risk of residual stenosis after PCB, including provisional stenting used for FPAD.

Subgroup analyses revealed several interesting outcomes. Firstly, the primary patency rate after PCB was lower in the subgroup of patients ≥ 70-years-old compared to those after PES. These findings suggested the PES should be applied to elderly patients with FPAD. Moreover, the improvement in the risk of TLR for patients with diabetes treated with PES might be superior to the PCB procedure, which could be explained by the findings that PES was suitable for high-risk patients. Finally, PES was superior to PCB for preventing death in patients with a lower current smoking proportion. This finding might effectuate the beneficial effects of antiplatelet drugs, as evident in smokers.

Nevertheless, the present study had some limitations. Firstly, this study was based on indirect comparative analysis, and the characteristics of patients were not balanced between the PCB and PES groups. Secondly, the number of trials that reported the effects of PCB and PES was not uniform, and the results from indirect comparisons might not be stable, the interpretation of the results in this study should be cautious. Thirdly, the heterogeneity among studies was not fully explained by subgroup analyses. Fourthly, there were inherent limitations in the meta-analysis, including restricted detailed analysis and inevitable publication bias, of note, clear publication bias found in most analyses. Finally, this meta-analysis was done based on study level which can inevitably lead to information loss; individual patient data was not obtainable and thus it was not performed.

This study did not find any significant differences between PCB and PES concerning the incidence of primary patency rate, TLR, death, restenosis, amputation, and thrombosis. The beneficial effects of PES were mainly detected in the groups of patients with mean age ≥ 70 years, diabetes proportion ≥ 40.0%, and current smoking < 60.0%. This finding in our study may provide hints for clinicians regarding the application of PES among specific patients. Therefore, further large-scale RCTs should be conducted to directly compare the treatment efficacy between PCB and PES in patients with FPAD.

In conclusion, PCB and PES are safe in treating FPAD. This study does not show the obvious advantage of PES in treating FPAD compared to PCB. We need more RCTs to confirm the differences in curative effects between PCB and PES.

## Author contributions

**Conceptualization:** Jie Fang.

**Data curation:** Jie Fang.

**Formal analysis:** Jie Fang.

**Funding acquisition:** Jie Fang.

**Investigation:** Yongbao Zhang.

**Methodology:** Jie Fang.

**Project administration:** Jie Fang.

**Resources:** Tingni Tang.

**Software:** Tingni Tang.

**Supervision:** Yongbao Zhang.

**Validation:** Jie Fang.

**Visualization:** Jie Fang.

**Writing – original draft:** Tingni Tang.

**Writing – review & editing:** Tingni Tang.

## Supplementary Material

SUPPLEMENTARY MATERIAL
