# Supplementary material for: Paclitaxel-coated balloon versus paclitaxel-eluting stent for femoropopliteal arterial disease: A meta-analysis
Source: Medicine (Baltimore). 2025 Mar 21;104(12):e41949. doi: 10.1097/MD.0000000000041949 (PMC11936668; doi:10.1097/MD.0000000000041949)

Supplemental Digital Content Figure S1. Pooled incidence for primary patency rate after treatment with PCB and PES.


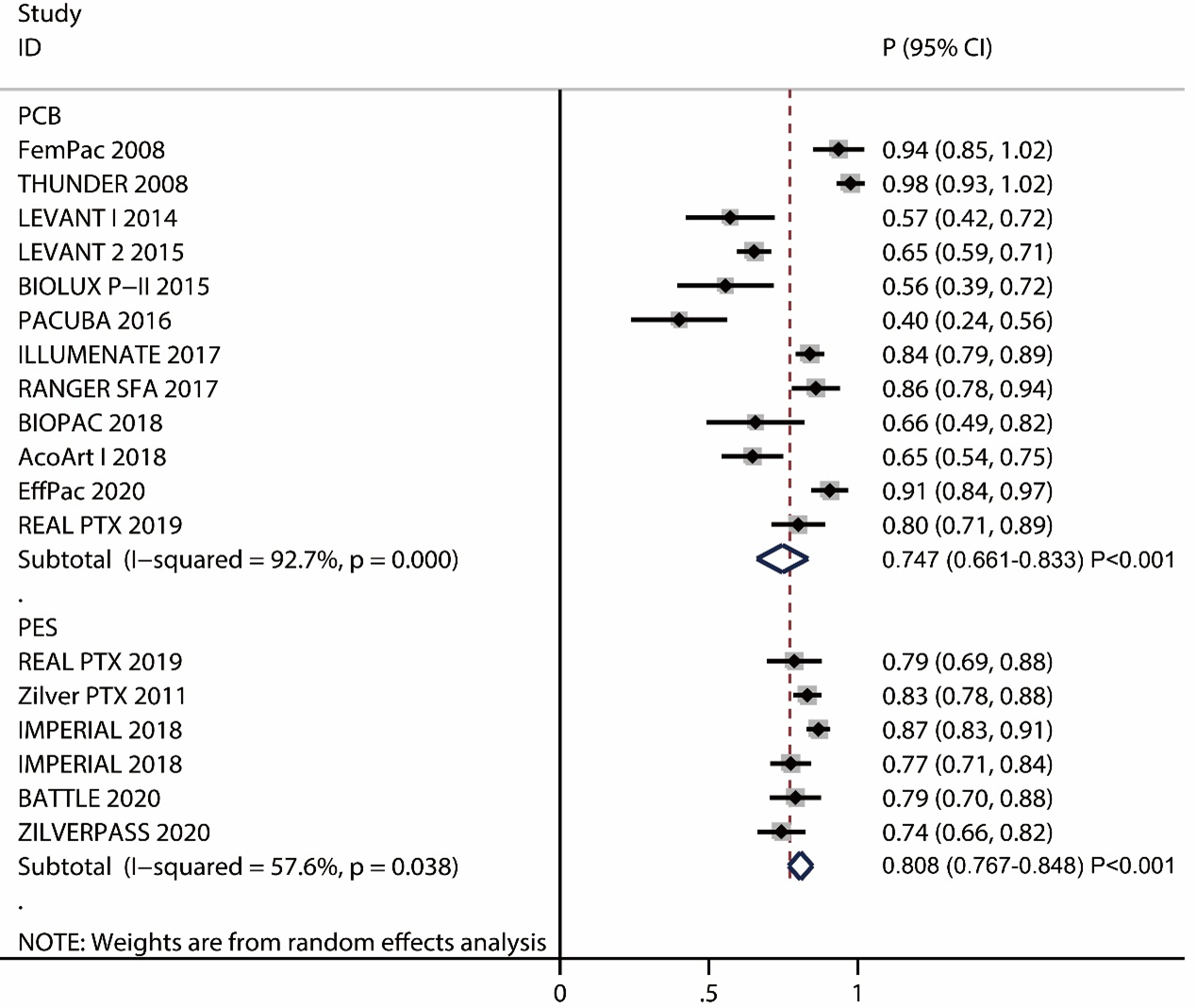


Supplemental Digital Content Figure S2. Funnel plot for primary patency rate


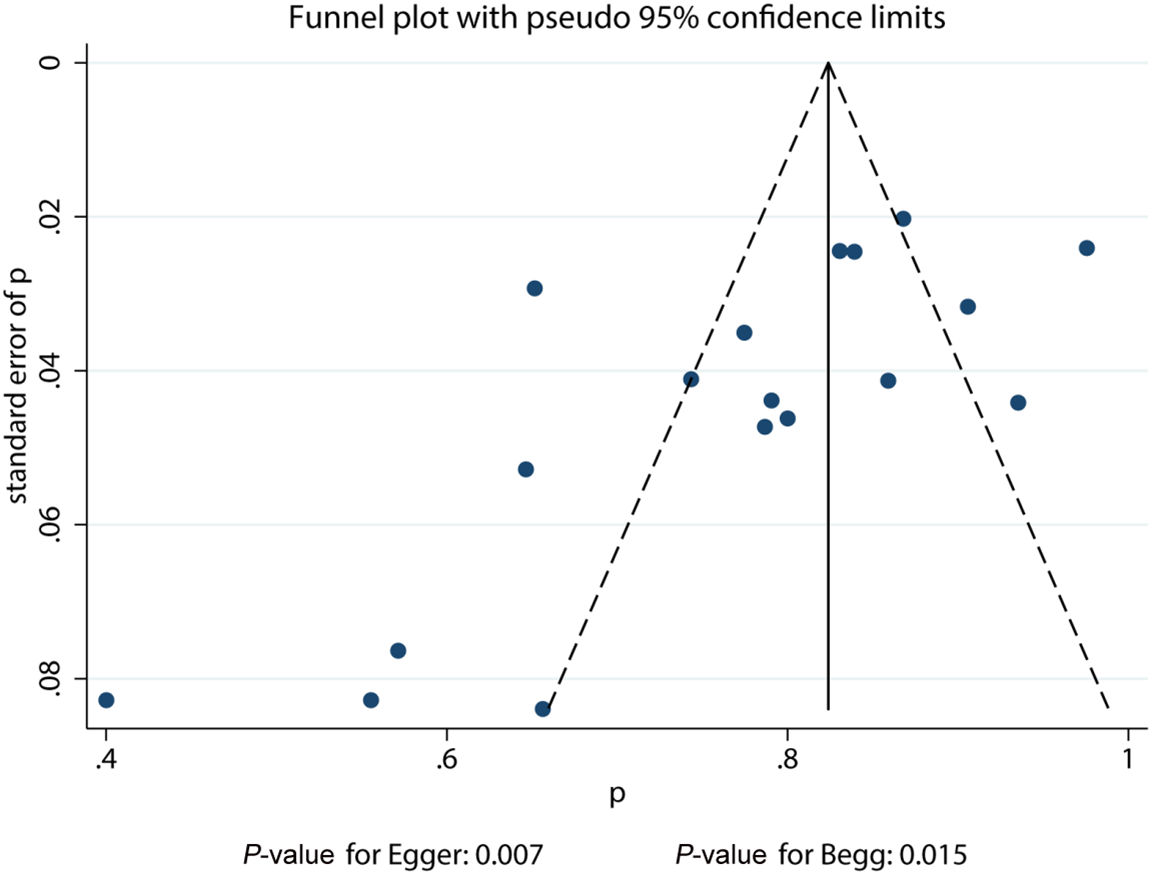


Supplemental Digital Content Figure S3. Funnel plot for TLR


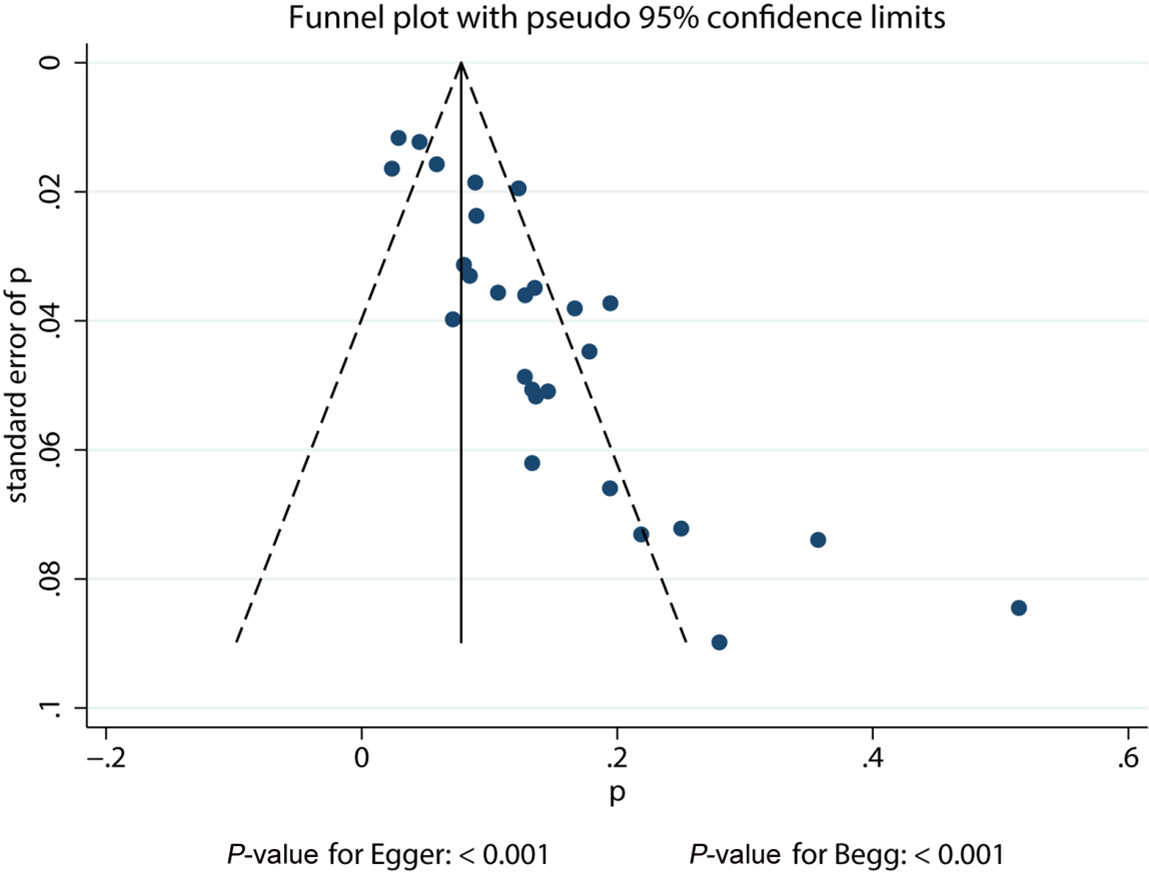


Supplemental Digital Content Figure S4. Funnel plot for death


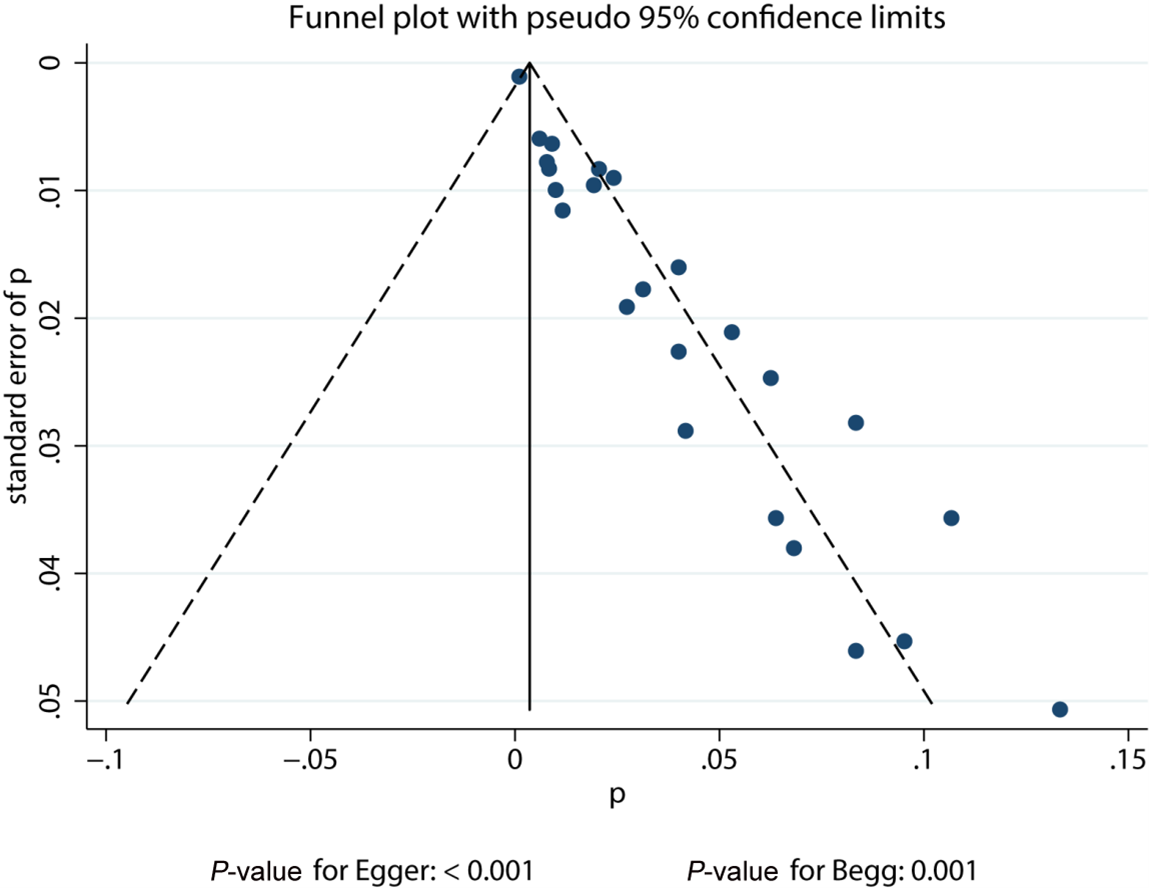


Supplemental Digital Content Figure S5. Funnel plot for restenosis


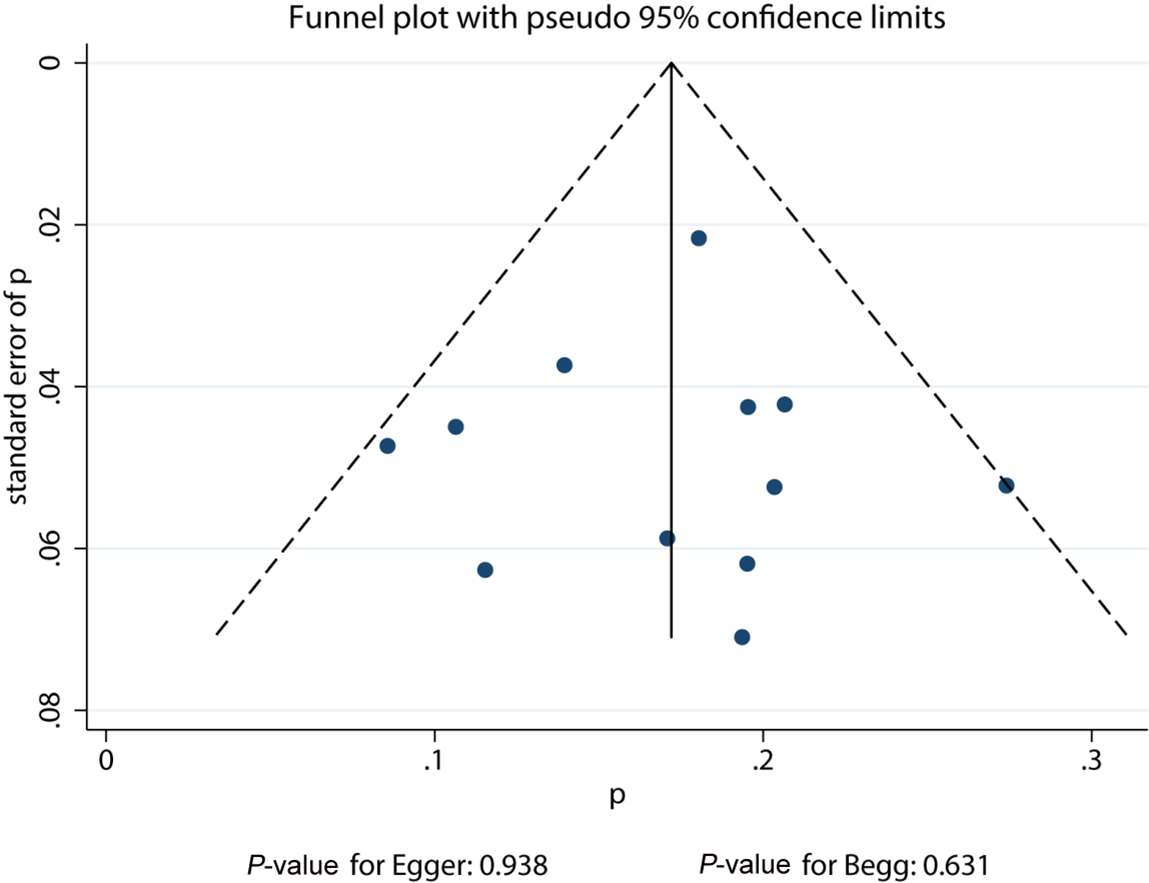


Supplemental Digital Content Figure S6. Funnel plot for amputation


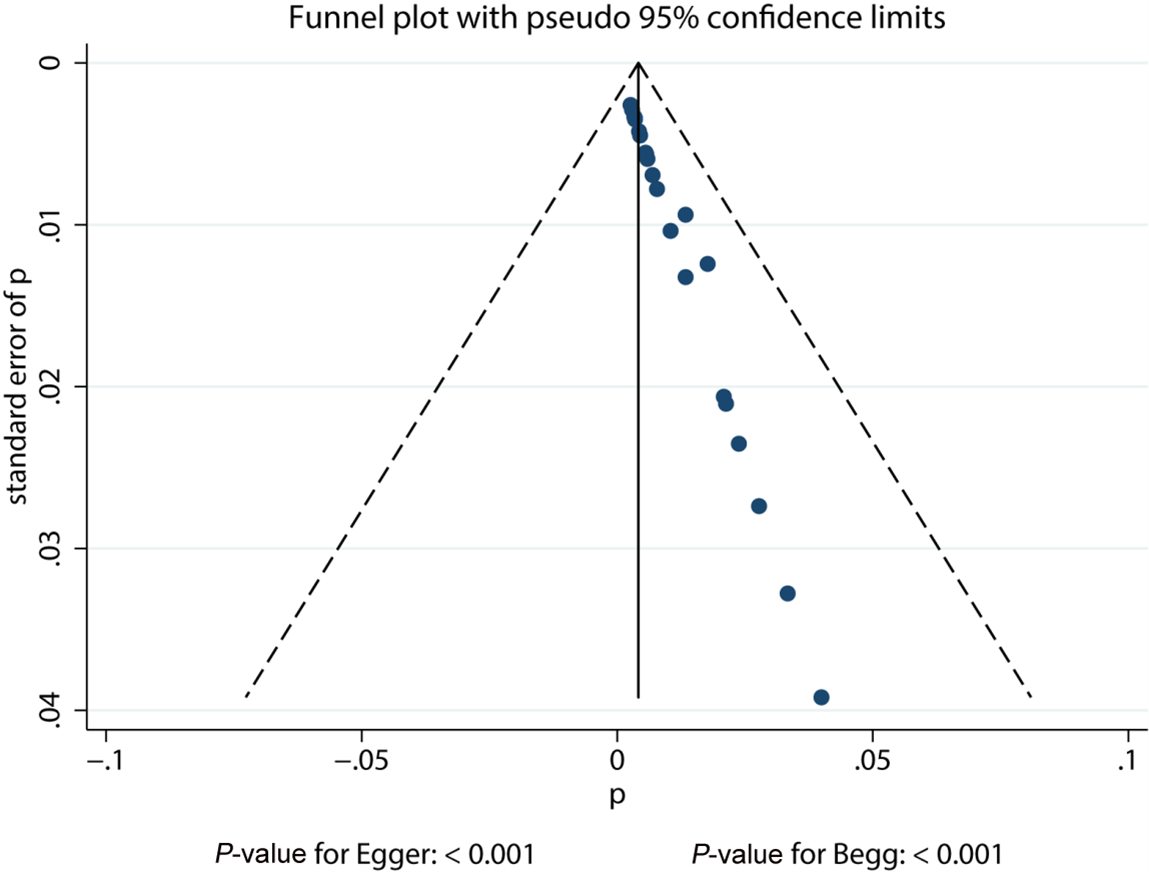


Supplemental Digital Content Figure S7. Funnel plot for thrombosis


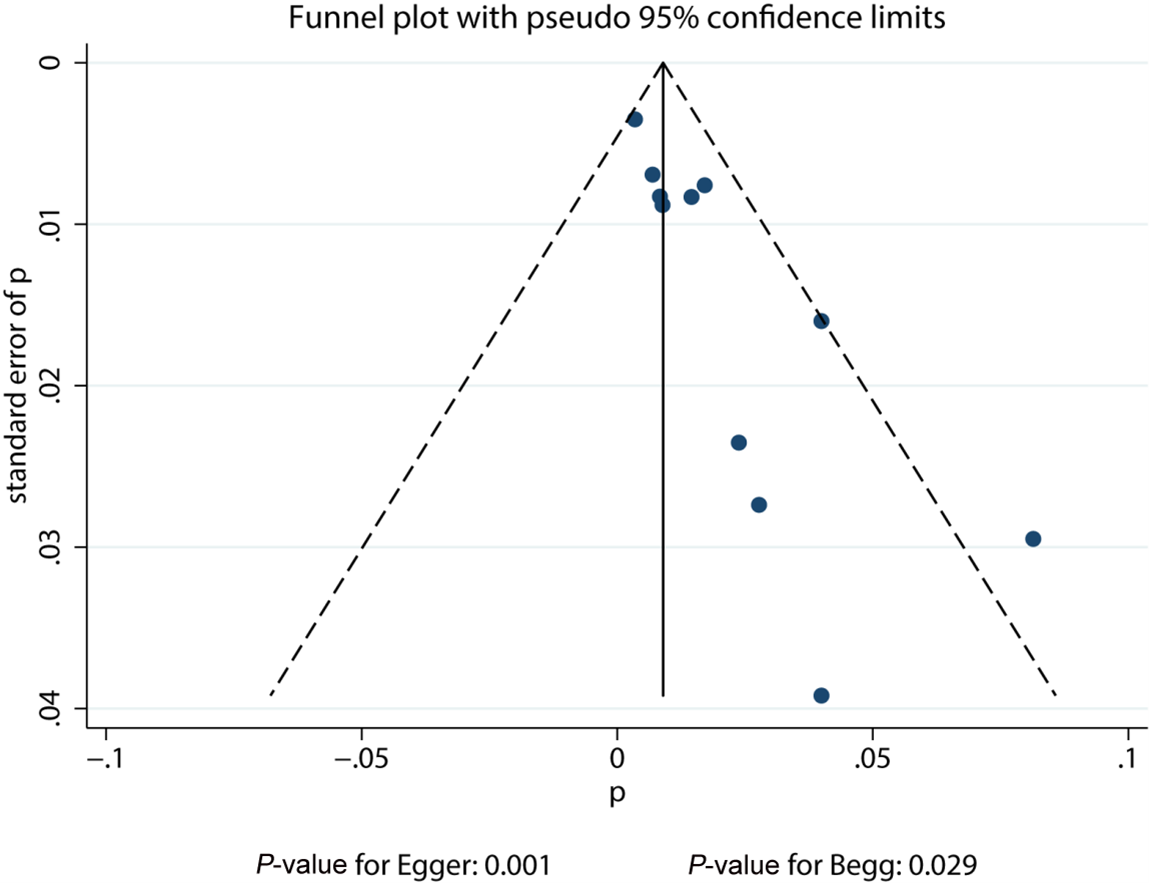

Supplement: SUPPLEMENTARY MATERIAL [file medi-104-e41949-s001.docx]
