# Supplementary material for: Paclitaxel-coated balloon versus paclitaxel-eluting stent for femoropopliteal arterial disease: A meta-analysis
Source: Medicine (Baltimore). 2025 Mar 21;104(12):e41949. doi: 10.1097/MD.0000000000041949 (PMC11936668; doi:10.1097/MD.0000000000041949)
Supplement: SUPPLEMENTARY MATERIAL [file medi-104-e41949-s002.pdf]

Supplemental Digital Content Table S1. Subgroup analysis for primary patency rate

**Variables**	**Groups**	**PCB**	**PES**	**RR and 95% CI**	**P-value**
Age (years)	≥ 70.0	0.556 (0.393–0.718)	0.791 (0.705–0.877)	**0.703 (0.510–0.968)**	**0.031**
	< 70.0	0.762 (0.675–0.850)	0.809 (0.763–0.855)	0.942 (0.828–1.071)	0.361
Male (%)	≥ 70.0	0.728 (0.621–0.836)	0.789 (0.726–0.852)	0.923 (0.779–1.092)	0.350
	< 70.0	0.760 (0.629–0.892)	0.812 (0.760–0.865)	0.936 (0.777–1.128)	0.486
Current smoking (%)	≥ 60.0	0.839 (0.791–0.887)	0.793 (0.709–0.878)	1.058 (0.937–1.194)	0.362
	< 60.0	0.742 (0.636–0.848)	0.813 (0.758–0.867)	0.913 (0.779–1.070)	0.259
Hypertension (%)	≥ 80.0	0.719 (0.579–0.859)	0.825 (0.781–0.868)	0.872 (0.711–1.069)	0.187
	< 80.0	0.781 (0.663–0.900)	0.765 (0.707–0.824)	1.021 (0.861–1.211)	0.812
Diabetes (%)	≥ 40.0	0.685 (0.526–0.844)	0.825 (0.782–0.867)	0.830 (0.652–1.058)	0.132
	< 40.0	0.837 (0.783–0.892)	0.762 (0.701–0.823)	1.098 (0.991–1.218)	0.075

Supplemental Digital Content Table S2. Subgroup analysis for TLR

Variables	Groups	PCB	PES	RR and 95% CI	P-value
Age (years)	≥ 70.0	0.136 (0.092–0.179)	0.146 (0.095–0.197)	0.932 (0.569–1.526)	0.778
	< 70.0	0.134 (0.093–0.174)	0.097 (0.055–0.139)	1.381 (0.790–2.417)	0.258
Male (%)	≥ 70.0	0.138 (0.082–0.194)	0.132 (0.091–0.174)	1.045 (0.610–1.792)	0.872
	< 70.0	0.137 (0.092–0.182)	0.096 (0.047–0.144)	1.427 (0.741–2.749)	0.288
Current smoking (%)	≥ 60.0	0.131 (0.017–0.246)	0.137 (0.034–0.240)	0.956 (0.183–5.005)	0.958
	< 60.0	0.137 (0.098–0.177)	0.100 (0.055–0.144)	1.370 (0.779–2.410)	0.275
Hypertension (%)	≥ 80.0	0.121 (0.071v0.171)	0.090 (0.054–0.127)	1.344 (0.728–2.482)	0.344
	< 80.0	0.145 (0.096–0.194)	0.161 (0.095–0.226)	0.901 (0.515–1.574)	0.713
Diabetes (%)	≥ 40.0	0.165 (0.111–0.219)	0.094 (0.056–0.133)	**1.755 (1.013–3.042)**	**0.045**
	< 40.0	0.097 (0.055–0.138)	0.150 (0.064–0.236)	0.647 (0.291–1.437)	0.284

Supplemental Digital Content Table S3. Subgroup analysis for death

Variables	Groups	PCB	PES	RR and 95% CI	P-value
Age (years)	≥ 70.0	0.027 (0.005–0.048)	0.017 (0.000–0.036)	1.588 (0.094–26.969)	0.749
	< 70.0	0.027 (0.014–0.040)	0.025 (0.004–0.046)	1.080 (0.286–4.080)	0.910
Male (%)	≥ 70.0	0.019 (0.003–0.036)	0.021 (0.004–0.038)	0.905 (0.169–4.838)	0.907
	< 70.0	0.031 (0.016–0.047)	0.023 (0.001–0.044)	1.348 (0.188–9.639)	0.766
Current smoking (%)	≥ 60.0	0.009 (0.000–0.018)	0.023 (0.000–0.073)	0.391 (0.010–15.987)	0.620
	< 60.0	0.045 (0.027–0.063)	0.023 (0.012–0.034)	**1.957 (1.000–3.828)**	**0.050**
Hypertension (%)	≥ 80.0	0.035 (0.016–0.053)	0.021 (0.003–0.040)	1.667 (0.400–6.943)	0.483
	< 80.0	0.025 (0.010–0.040)	0.029 (0.000–0.069)	0.862 (0.043–17.365)	0.923
Diabetes (%)	≥ 40.0	0.035 (0.019–0.051)	0.017 (0.001–0.032)	2.059 (0.340–12.478)	0.432

< 40.0	0.016 (0.002–0.030)	0.047 (0.017–0.077)	0.340 (0.072–1.605)	0.173

Supplemental Digital Content Table S4. Subgroup analysis for restenosis

Variables	Groups	PCB	PES	RR and 95% CI	P-value
Age (years)	≥ 70.0	0.149 (0.091–0.206)	0.170 (0.105–0.236)	0.876 (0.493–1.558)	0.653
	< 70.0	0.183 (0.144–0.222)	-	-	-
Male (%)	≥ 70.0	0.195 (0.074–0.316)	0.170 (0.105–0.236)	0.147 (0.500–2.634)	0.746
	< 70.0	0.170 (0.135–0.205)	-	-	-
Current smoking (%)	≥ 60.0	0.115 (0.000–0.238)	-	-	-
	< 60.0	0.175 (0.142–0.209)	0.170 (0.105–0.236)	1.029 (0.657–1.612)	0.899
Hypertension (%)	≥ 80.0	0.173 (0.139–0.207)	0.207 (0.124–0.289)	0.836 (0.524–1.334)	0.452
	< 80.0	0.173 (0.116–0.229)	0.140 (0.066–0.213)	1.236 (0.628–2.433)	0.540
Diabetes (%)	≥ 40.0	0.165 (0.136–0.195)	0.170 (0.105–0.236)	0.971 (0.623–1.512)	0.895

	< 40.0	0.203 (0.116–0.289)	-	-	-

Supplemental Digital Content Table S5. Subgroup analysis for amputation

Variables	Groups	PCB	PES	RR and 95% CI	P-value
Age (years)	≥ 70.0	0.004 (0.000–0.008)	0.003 (0.000–0.007)	1.333 (0.103–17.276)	0.826
	< 70.0	0.005 (0.001–0.008)	0.005 (0.000–0.010)	1.000 (0.109–9.163)	1.000
Male (%)	≥ 70.0	0.006 (0.000–0.012)	0.003 (0.000–0.007)	2.000 (0.133–30.097)	0.616
	< 70.0	0.004 (0.001–0.007)	0.005 (0.000–0.010)	0.800 (0.090–7.110)	0.841
Current smoking	≥ 60.0	0.005 (0.000–0.014)	0.006 (0.000–0.015)	0.833 (0.040–17.224)	0.906
(%)	< 60.0	0.004 (0.001–0.007)	0.003 (0.000–0.007)	1.333 (0.176–10.117)	0.781
Hypertension (%)	≥ 80.0	0.005 (0.000–0.011)	0.004 (0.000–0.007)	1.250 (0.086–18.206)	0.870
	< 80.0	0.004 (0.001–0.007)	0.005 (0.000–0.016)	0.800 (0.089–7.155)	0.842
Diabetes (%)	≥ 40.0	0.004 (0.001–0.008)	0.003 (0.000–0.006)	1.333 (0.182–9.786)	0.777
	< 40.0	0.005 (0.000–0.009)	0.016 (0.000–0.033)	0.313 (0.013–7.548)	0.474
	< 80.0	0.010 (0.000–0.026)	0.040 (0.000–0.110)	0.250 (0.005–13.441)	0.495

Diabetes (%)	≥ 40.0	0.006 (0.000–0.012)	0.037 (0.006–0.067)	0.162 (0.015–1.746)	0.133
	< 40.0	0.010 (0.000–0.026)	0.009 (0.000–0.026)	1.111 (0.036–34.717)	0.952

Supplemental Digital Content Table S6. Subgroup analysis for thrombosis

Variables	Groups	PCB	PES	RR and 95% CI	P-value
Age (years)	≥ 70.0	0.008 (0.000–0.018)	0.081 (0.024–0.139)	0.099 (0.009–1.105)	0.060
	< 70.0	0.006 (0.000–0.012)	0.018 (0.004–0.031)	0.333 (0.034–3.288)	0.347
Male (%)	≥ 70.0	0.008 (0.000–0.021)	0.081 (0.024–0.139)	0.099 (0.008–1.188)	0.068
	< 70.0	0.006 (0.000–0.012)	0.018 (0.004–0.031)	0.333 (0.034–3.288)	0.347
Current smoking (%)	≥ 60.0	0.010 (0.000–0.026)	0.009 (0.000–0.026)	1.111 (0.036–34.717)	0.952
	< 60.0	0.006 (0.000–0.012)	0.037 (0.006–0.067)	0.162 (0.015–1.746)	0.133
Hypertension (%)	≥ 80.0	0.006 (0.000–0.012)	0.024 (0.003–0.045)	0.250 (0.021–2.910)	0.268
	< 80.0	0.010 (0.000–0.026)	0.040 (0.000–0.110)	0.250 (0.005–13.441)	0.495
Diabetes (%)	≥ 40.0	0.006 (0.000–0.012)	0.037 (0.006–0.067)	0.162 (0.015–1.746)	0.133

< 40.0	0.010 (0.000–0.026)	0.009 (0.000–0.026)	1.111 (0.036–34.717)	0.952
